# BarraCUDA - a fast short read sequence aligner using graphics processing units

**DOI:** 10.1186/1756-0500-5-27

**Published:** 2012-01-13

**Authors:** Petr Klus, Simon Lam, Dag Lyberg, Ming Sin Cheung, Graham Pullan, Ian McFarlane, Giles SH Yeo, Brian YH Lam

**Affiliations:** 1 University of Cambridge Metabolic Research Laboratories, Institute of Metabolic Science, Box 289, Addenbrooke's Hospital, Hill's Road, Cambridge CB2 0QQ, UK; 2 Genomics CoreLab, NIHR-Cambridge Biomedical Research Centre, Box 232, Addenbrooke's Hospital, Hill's Road, Cambridge CB2 0QQ, UK; 3 Department of Microbiology, University College Cork, College Road, Cork, Ireland; 4 The Gurdon Institute, University of Cambridge, Tennis Court Road, Cambridge CB2 1QN, UK; 5 Whittle Laboratory, University of Cambridge, JJ Thomson Avenue, Cambridge CB3 0DY, UK

## Abstract

**Background:**

With the maturation of next-generation DNA sequencing (NGS) technologies, the throughput of DNA sequencing reads has soared to over 600 gigabases from a single instrument run. General purpose computing on graphics processing units (GPGPU), extracts the computing power from hundreds of parallel stream processors within graphics processing cores and provides a cost-effective and energy efficient alternative to traditional high-performance computing (HPC) clusters. In this article, we describe the implementation of BarraCUDA, a GPGPU sequence alignment software that is based on BWA, to accelerate the alignment of sequencing reads generated by these instruments to a reference DNA sequence.

**Findings:**

Using the NVIDIA Compute Unified Device Architecture (CUDA) software development environment, we ported the most computational-intensive alignment component of BWA to GPU to take advantage of the massive parallelism. As a result, BarraCUDA offers a magnitude of performance boost in alignment throughput when compared to a CPU core while delivering the same level of alignment fidelity. The software is also capable of supporting multiple CUDA devices in parallel to further accelerate the alignment throughput.

**Conclusions:**

BarraCUDA is designed to take advantage of the parallelism of GPU to accelerate the alignment of millions of sequencing reads generated by NGS instruments. By doing this, we could, at least in part streamline the current bioinformatics pipeline such that the wider scientific community could benefit from the sequencing technology.

BarraCUDA is currently available from http://seqbarracuda.sf.net

## Background

Next-generation sequencing (NGS) is a technique based on sequencing by synthesis or sequencing by ligation in a massively parallel fashion and generates short sequencing reads ranging from 25 to 400 bp. The first commercially available next-generation sequencer, the Genome Sequencer 20 was released by 454 Life Sciences in 2005 [1] with the hope of enabling the analysis of complete genomes within a short period of time [2]. It produced a throughput of 20 megabases from a 5-hour run, which was 30 fold higher than traditional Sanger capillary electrophoresis systems. Over these years, the output of next-generation sequencers has increased 30, 000 fold to 600 gigabases from a single instrument run (Illumina Hiseq 2000) which is about 200 times the depth of coverage of an entire human genome.

The advancement of the technology has generated an enormous amount of sequence data [3]. Sequence alignment is one of the first steps for downstream data analyses, during which sequencing reads have to be mapped either to other reads to form a genome (also known as *de novo *sequence assembly) [1,4]; or on to a reference DNA sequence, usually a genome, for downstream applications such as single-nucleotide polymorphism (SNP) discovery [3], Chip-Seq [5] or RNA-Seq [6]. Here we focus on the latter type of alignments.

A handful of software packages have been developed for computing alignments of sequencing reads generated by NGS instruments on to a reference sequence. Early generation aligners such as MAQ [7], RMAP [8,9] and Soap [10] use hash-based algorithms to perform the mapping of sequencing reads on to reference genomes. Even though these tools can be accelerated by several seeding approaches, or parallelized by using multiple cores and cloud computing (e.g. CloudBurst [11]), the computational cost remains expensive. For example, by extrapolating the result from Schtaz et al. [11], it would take over 30, 000 CPU hours to align reads generated from a single HiSeq 2000 run.

Later in 2009, a new generation of sequence aligners were released, namely Soap2 [12], Bowtie [13] and BWA [14]. These tools use a suffix tree-based algorithm (also known as FM-index) based on Burrows-Wheeler Transform (BWT). BWT is a block-sorting algorithm originally designed for lossless data compression [15], it can also be used for string matching by a backward search approach [16]. The major advantages of this approach include a low time complexity of *O*(*n*) to find an exact match where *n *is the length of the query [16] and the performance is independent from the size of the reference sequence. In addition, a high compression ratio of 2.5 bit per base for the reference genome [17] also means a full human genome can fit into 1.3 GB of space. The new algorithm is a magnitude quicker and much more memory efficient than their hash-based predecessors [14]. In an experiment performed by Li and Durbin [14] the alignment throughput for 12.2 million 51 bp reads being mapped to the Human genome went down from 94 CPU hours (MAQ) to 4 CPU hours (BWA) on a 2.5 GHz Intel Harpertown-based Xeon E5420 while retaining comparable accuracy in alignment mapping.

Modern graphics cards are designed primarily for rendering real time, high-definition complex 3D graphics features for various visual applications such as gaming and graphics design. Each graphics processing unit, or GPU, consists of many high performance stream processors capable of performing billions of independent calculations per second in order to meet the high visualization demand required for graphics applications. It is this processing capability that can be translated into a general-purpose computation capability equivalent to a small cluster of traditional CPUs. In addition, the lower energy profile and cost means the use of GPU to perform parallel computing tasks has become increasingly attractive. Many modern supercomputers including the Chinese Tianhe-1A, Nebulae and Japanese Tsubame 2.0 (http://www.top500.org/lists/2010/11) also contain multiple GPU nodes on top of traditional nodes with CPUs to take advantage to the parallel computing capability of GPUs.

MUMmerGPU [18,19] is one of the first GPGPU-based DNA alignment software that utilizes the NVIDIA Compute Unified Device Architecture (CUDA) to perform variable-length maximal exact DNA alignments. Unlike other BWT aligners it uses a different suffix-tree approach, namely Ukkonen's algorithm [20] to find exact matches in the reference sequence of all the sub-strings in the query DNA sequence. The current version of MUMmer GPU out-performs its CPU counterpart by 13 fold [18,19]. However, unlike other sequence aligners mentioned in the previous section, MUMmerGPU does not support inexact alignments by itself and has to be used in conjunction with the original MUMmer software package [21] to perform inexact alignments.

Here we introduce BarraCUDA, a program that takes advantage of GPGPU to perform inexact alignment of sequencing reads on to a reference sequence. BarraCUDA is built on the foundation of BWA and we have rewritten the BWT-based alignment core module to make use of the massive parallelism of GPGPU. It also employs the fast and memory efficient BWT-based algorithm employed in the original software and supports mismatches and full gapped alignment of sequencing reads.

### Software implementation

BarraCUDA utilises NVIDIA's GPGPU implementation, namely Compute Unified Device Architecture (CUDA) to parallelise the alignment of sequence reads. Firstly, the program loads the complete BWT-encoded reference sequence and sequence reads from disk to GPU memory; This is followed by launching a GPU alignment kernel, where the alignment task of each of the sequence reads are distributed to hundreds of processors within the GPU and computations are performed in parallel; Once the kernel finishes, the alignment results are transferred from GPU back to disk. The following sections describe the details of each of the steps performed in BarraCUDA. (Please also refer to the Additional file 1 for the pseudo-code algorithm framework)

#### 1. Transferring BWT-encoded reference sequence and sequence reads from disk to GPU

BarraCUDA first loads the full BWT suffix array from disk into cached texture memory in the GPU using a 1-dimensional *uint4 *array to ensure fast data access. Sequence reads are loaded into GPU memory in batches and packed in a single continuous block to minimise internal fragmentations, and the data is also bound to the texture cache to maximise the data throughput.

#### 2. CUDA thread assignments

Mapping a sequence read to a reference sequence is a data independent process and does not require any information from any of the other reads, thus BarraCUDA employs a straightforward data parallelism by assigning an alignment kernel thread to each of the individual sequencing reads and launching the GPU kernel with tens of thousands of threads at the same time.

#### 3. Inexact sequence alignment--a depth-first search GPU kernel

**Figure 1:**
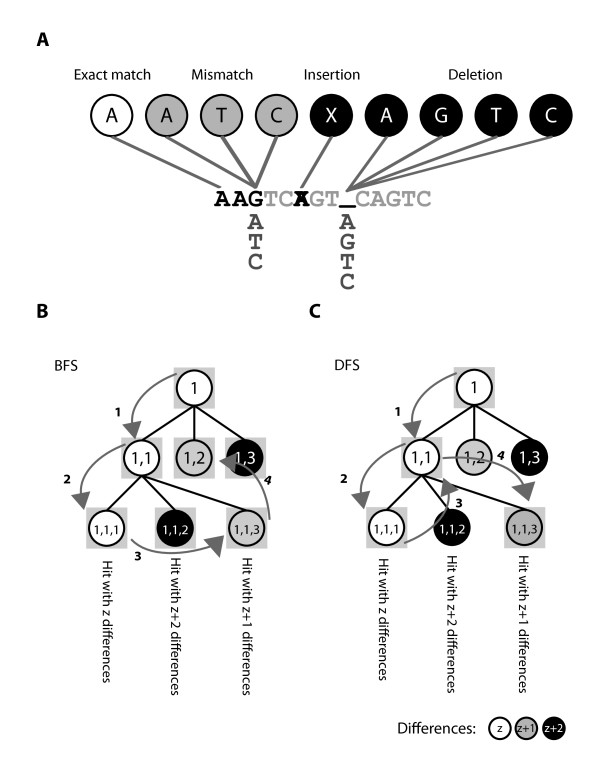
**The search space traversal for inexact alignments**. **A**. There are 4 types of alignments for each base, namely exact match, mismatch, insertion and deletion. A mismatch alignment is performed by substituting the reference base G with the three other possible bases (A, T and C). Insertions are detected by deleting the base from the query sequence, and deletions by inserting all 4 bases into the query sequence. **B**. A BFS strategy: BFS starts from the first base (1) and stores all hits in daughter nodes (1, 1)...(1, 3) in memory (with shaded squares), then it chooses (1, 1) and expands it into (1, 1, 1) ... (1, 1, 3). With all the nodes in memory, the agent then evaluates (1, 1, 1) which returns an alignment followed by (1, 1, 3), a suboptimal alignment, which is the next best hit in memory. After that the agent proceeds to (1, 2) which has the same number of differences as (1, 1, 3). BWA does not process nodes with more than z + 1 differences, i.e. (1, 3), (1, 1, 2) with the default option. **C**. A DFS strategy: DFS chooses the best hit (1, 1) from (1), and subsequently chooses (1, 1, 1) which returns an alignment. Then the agent goes back to (1, 1) to reach (1, 1, 3) as the next best hit to return the sub-optimal alignment. After that, the agent returns to (1, 1), then (1) to reach (1, 2) (not shown). Like BWA, BarraCUDA skips nodes with > z + 1 differences by default.

The alignment kernel in BarraCUDA, like BWA and all other BWT-based sequence aligners, consists of a backward search string-matching algorithm [13,14] to look for alignments. Inexact alignment requires a search space of *O*(9*^n^*) in order to generate and evaluate a series of base substitutions that could lead to an exact string match (Figure 1a). BarraCUDA differs from BWA in its strategy to perform search traversal. While BWA utilises a time efficient breadth-first search (BFS) approach as shown in Figure 1b, it can utilize a maximum of 40 MB of memory space for each computational thread (please refer to the Additional file 1 for explanations). With thousands of concurrent threads on the GPU, the memory to each thread is very limited and BFS does not seem to be an option. Therefore in BarraCUDA, we adopted a difference-bound DFS approach (as outlined in Figure 1c) where it uses 20, 000 fold less memory to perform alignments (see Additional file 1). Rather than storing all partial hits while traversing through the search space, the DFS algorithm only stores in memory the branch of the search space with the local best hit score, i.e. (1), (1, 1) followed by (1, 1, 1) to give a full alignment (Figure 1c). Nonetheless, DFS is not as time efficient as BFS employed in BWA, BarraCUDA has to re-assess nodes multiple times until all possible hits from that node are evaluated, for instance in Figure 1c, the program has to return from (1, 1, 1) to (1, 1) in order to reach (1, 1, 3) as the next best hit, and this is particular a problem when the read length is long and the search space is extremely large.

#### 4. Multiple kernel design

**Figure 2:**
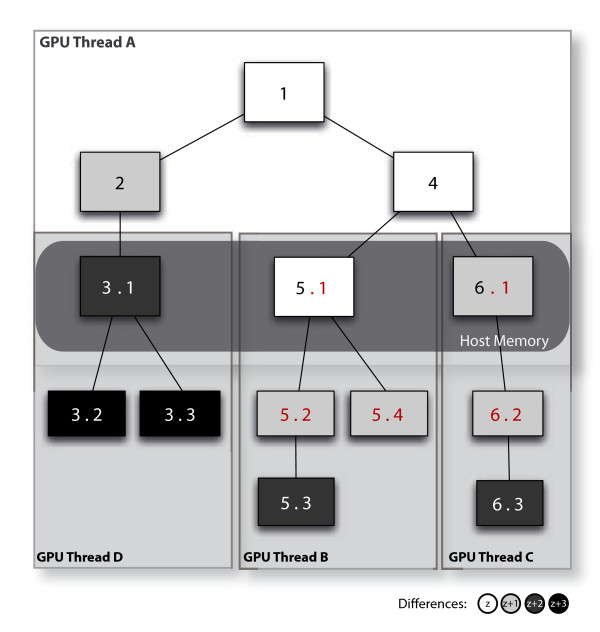
**A multiple kernel design**. The alignment of a sequencing read is performed by multiple DFS kernel threads. Each of the sequencing reads is partitioned into short fragments and multiple kernel threads are launched to map each of the fragments to the reference in a consecutive manner. The figure shows an example of mapping a 5 bp read: The first kernel thread (A) processes only the first 3 bp and returns partial hits (3), (5) and (6) to a temporary memory store in the host computer, ranked according to the number of differences in these hits, mimicking the BFS strategy in BWA. Following that, threads (B), (C) and (D) are launched in an orderly manner to map the remaining 2 bp to obtain the full alignments (5, 3) and (6, 3).

Long sequence reads are divided into short fragments 32 bp (default seed length) and alignment is performed by multiple consecutive DFS kernel runs. Figure 2 illustrates an example of such approach with the alignment of a 5 bp read, the first DFS kernel (GPU thread A) only maps up to the third base (nodes 3.1, 5.1 and 6.1), and partial alignments at this point are returned from the GPU and stored in a temporary memory stack on the host computer (rounded square). After that, similar to BWA's BFS, all partial hits in the host memory store are ranked by their number of differences by the host code where the best hits are prioritised for the subsequent kernel launch, (i.e. GPU thread B, GPU thread C followed by GPU thread D). By doing this, we could reduce significantly the number of revisits by the DFS agent to the length of the fragments (32 bp), and thus lower the serialisations caused by thread divergence compared to alignment using one single kernel. In addition, this could also allow us to discard partial hits that have more than *z *+ 1 differences on the memory stack, as in BWA, when a full alignment is found, to increase the computing efficiency.

#### 5. Alignment data management

During runtime, the alignment data for each of the sequence reads including BWT suffix array (SA) coordinates and the number of differences is stored temporarily in GPU memory. Once the kernel finishes, the data are copied from the GPU back to the host and subsequently written onto disk storage in a binary file format. Similar to BWA, the SA coordinates can then be translated to linear space using BarraCUDA's 'samse' or 'sampe' cores for single-end or paired-end libraries respectively.

## Findings

### BarraCUDA shares a similar alignment accuracy as BWA

It is important that the changes in the search algorithm and alignment criteria in BarraCUDA do not penalize the alignment accuracy of the software. To test this, we first generated a library of 1 million pairs of simulated reads of 70 bp from the *Caenorhabditis Elegans *genome (WS200.55, 102 million bp) using 'wgsim' from SAMtools [22] with a base error rate of 0.02. We then mapped the reads back to the same genome using BWA and BarraCUDA. To measure the mapping accuracy we calculated the percentage of reads mapped, and the rate of incorrect mappings using a mapping quality threshold of 10 (Phred-scale). As shown in Table 1, both BWA and BarraCUDA reported a similar percentage of reads mapped to the *C. Elegans *genome at around 90% and 96% single-end and paired-end respectively. The error rate was also very similar between BWA and BarraCUDA, where about 0.06% of both single and paired end reads were incorrectly aligned to the genome.

**Table 1:** Alignment accuracy compared to BWA using simulated reads

	BWA		BarraCUDA	
	
	Gap enabled	Gap disabled	Gap enabled	Gap disabled
**Single-end**				

% Mapped	89.95	91.01	91.42	91.14

% Error	0.06	0.04	0.05	0.05

**Paired-end**				

% Mapped	96.53	96.50	96.61	96.64

% Error	0.04	0.04	0.06	0.06

### Ungapped alignment has minimal effects on alignment accuracy

Gapped alignment is costly in terms of alignment throughput, due to the much larger search space *O*(9*^n^*) compared to *O*(4*^n^*) when gap opening is disabled (Figure 1a). In a separate experiment, we performed the alignment of the same set of data above with gap opening disabled (using option '-o 0') and found that the number of confident mappings and the error rates were largely unaffected (Table 1).

The choice between gapped and ungapped alignment is largely dependent on the nature of the sequencing experiments. For re-sequencing studies, gapped alignment is essential to minimise false positive variant calls [23]. On the other hand, we would recommend disabling gap opening using option '-o 0' for experiments such as Chip-seq or RNA-seq for good performance.

### Gapped alignment throughput of a GPU is equivalent to that of 6 CPU cores

The alignment throughputs of BarraCUDA and BWA were measured by mapping 2 sets of paired-end whole-genome shotgun libraries containing sequencing reads of 37 bp, and 76 bp in length (1000 Genomes Project, European Nucleotide Archive accession: ERR003014 and SRR032215 respectively) on to the human genome (NCBI36.54).

For BWT-index construction, both BWA and BarraCUDA utilize the same BWT-indexing core [24] and took about 1.5 h to complete (data not shown). The encoded genome was 2.6 GB including both the forward and reverse BWT indices. It is useful to note that all BWT-related files (.bwt,.rbwt,.ann,.sa,.rsa,.pac,.rpac) only needed to be generated once, and that the files are compatible between the two programs.

**Figure 3:**
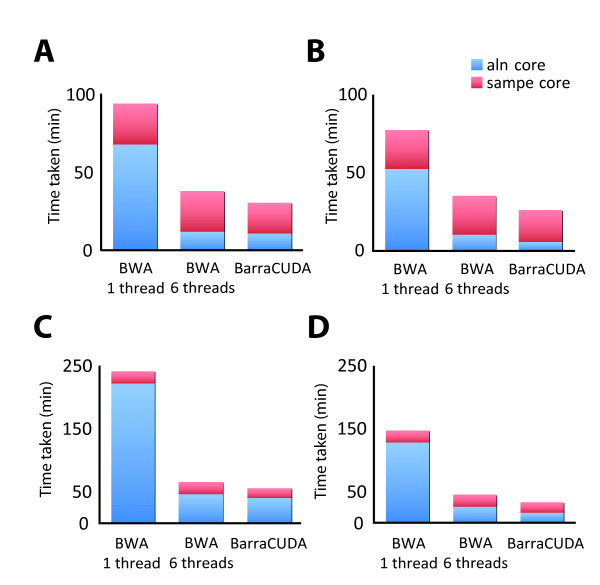
**A comparison of alignment throughput of BWA and BarraCUDA in align real-life sequencing reads to the human genome**. Two whole-genome shotgun libraries from the 1000 Genomes Project were used to compare the paired-end alignment throughput between BWA and BarraCUDA. **A**. 11.3 million pairs of 37 bp reads (ENA accession: ERR003014) were aligned to the human genome (NCBI 36.54) using BWA v0.5.8 with a server class Intel Xeon 5670 (utilising 1 or 6 threads) and BarraCUDA with an NVIDIA Tesla M2090, both with default options. The figure shows the time taken for 'aln' core (in blue) and 'sampe' core (in red); **B**. The time taken with gap opening disabled using the option '-o 0'; **C**. The time taken to align 14.5 million pairs of 76 bp reads (ENA accession: SRR032215) using the same set of hardware; **D**. The timings with gap opening disabled.

Figure 3a and 3b depicts the relative alignment throughputs (including the alignment 'aln' core in blue and SAM output 'sampe' core in red) for the 37 bp read library of BWA and BarraCUDA respectively. For gapped alignments, running BWA alignment core with an Intel Westmere-based Xeon X5670 2.93 GHz CPU and 8 GB DDR3 memory with 1 thread took 67 m 56 s to map all the 11.3 million pairs of reads in the library, while the time taken was reduced to 11 m 51 s when the same task was performed using all 6 cores on the CPU (Figure 3a). On the other hand, BarraCUDA took 10 m 51 s to perform the same task using an NVIDIA Tesla M2090, which was on a par with BWA using all 6 cores on the X5670.

For ungapped alignments, while BWA exhibited a speed increase of 14.6% with 6 threads, we observed a 2.1 fold speed-up in BarraCUDA compared to gapped alignment (Figure 3b). In this experiment, BarraCUDA only took 5 m 46 s to align all 11.3 million pairs of read onto the human genome, almost half the time taken by BWA with 6 threads.

The SAM conversion 'samse' and 'sampe' cores in BWA, which convert alignments coordinates from BWT SA intervals back to linear space on the reference genome, are comparative less computational intensive than the alignment core as seen in Figure 3 and thus do not make use of multiple threads. We did not port the 'samse/sampe' to GPU in this version of BarraCUDA, but we improved the conversion speed by an average of 27% through the use of a more efficient memory management strategy in working with BWT indices.

With increased number of reads and a longer read length when aligning the 76 bp library, both software took a longer time to complete the mappings (Figure 3c). BWA with a single thread took almost 4 h to complete the gapped alignment of 14 million pairs of sequencing reads in the library, it was significantly shortened to 46 m 10 s when all 6 cores on the X5670 were used. BarraCUDA took 40 m 1 s to complete the alignment that was again similar to BWA with 6 threads.

When gap opening was disabled, the throughput of BWA was doubled (Figure 3d). Similarly, the time taken for BarraCUDA was also significantly shortened when gapped alignment was disabled, to 16 m 21 s which is 56% faster than BWA with 6 threads.

The 'sampe' core, on the other hand was not affected by the size and the read length of the library and took roughly the same time as the 37 bp library to convert the alignment coordinates. Again, BarraCUDA version of 'sampe' was slightly faster than BWA in this test.

### Percentage of mappings

The percentage of confident mappings was similar between BWA and BarraCUDA (Table 2), and like the artificial *C. Elegans *data set, the effect on the percentage of mapping by disabling gapped alignments was minimal.

**Table 2:** The percentage of confident mappings between BWA and BarraCUDA

	BWA		BarraCUDA	
	
	Gap enabled	Gap disabled	Gap enabled	Gap disabled
**37 bp library**				

% Mapped	77.19	76.91	77.16	76.99

**76 bp library**				

% Mapped	82.74	82.87	82.75	82.58

### Multiple GPU configuration

For computers with multiple CUDA-capable GPUs, BarraCUDA automatically selects the best GPU based on number of stream processors and the amount of graphics memory available to the software. Users can also specify which CUDA device the software is to be executed on by using the '-C' option followed by the device number. In order to take advantage of multiple GPUs in a system, BarraCUDA is accompanied with two scripts, namely 'barracuda-multi-se' and 'barracuda-multi-pe' to align parallel single-end reads and paired-end reads respectively using multiple GPUs. 'barracuda-multi-se' automatically detects the number of CUDA devices in the computer, splits the input.fastq read files according to the number of CUDA devices and calls multiple instances of BarraCUDA to align sequencing reads ('aln' and 'samse') in parallel. Once the alignment finishes, the script joins the files back into one single SAM file. For paired-end reads, 'barracuda-multi-pe' calls two instances of BarraCUDA to align the two paired.fastq read files at the same time and generates a single SAM output using the 'sampe' core. At the time of writing, 'barracuda-multi-pe' does not support more than 2 GPUs while 'barracuda-multi-se' is not bounded by the number of CUDA devices.

### Multiple GPUs show a better scalability than CPUs

**Figure 4:**
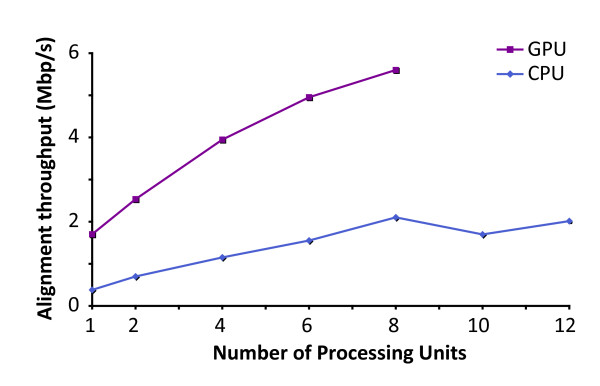
**The scalability of alignment throughputs using multiple GPUs and CPUs**. This figures shows the effect on alignment throughputs (in megabases per seconds, Mbp/s) when multiple GPUs and CPUs were used to map a single-end library containing 13.6 million 96 bp reads to the D. Melanogaster genome. A computer node containing two 6-core Intel Xeon 5670 s and eight NVIDIA Tesla M2050 were used in this test. The throughputs of BWA were measured with 1, 2, 4, 6, 8, 10 and 12 threads and BarraCUDA with 1, 2, 4, 6 and 8 M2050s using default options.

Figure 4 shows the scalability of using multiple GPUs and CPUs in aligning another whole-genome shotgun library of 13.5 million single-end 95 bp reads (ENA accession: SRR063699) to the *Drosophila Melanogaster *genome (BDGP5.25.63). Similar to the human library we examined earlier, the alignment throughput of BarraCUDA with 1 Tesla M2050 GPU was similar to that of BWA with 6 CPU cores (Xeon X5670 2.93 GHz with 8 GB DDR3 RAM). We tried to boost further the speed of BWA with more CPU cores, but we did not find any additional benefit beyond 8 cores. On the other hand we found that using BarraCUDA with two GPUs already outperformed BWA using all 12 cores (2× Xeon X5670s) at 2.5 Mbp/s, and the alignment throughput when used with 8 GPUs took only 3.8 min, which was 2.8 times the speed of BWA utilising all 12 CPU cores available on the computer node. The difference in the scalabilities between CPUs and GPUs is mainly due to the difference in memory bandwidths, where each GPU has exclusive access to their own dedicated on-board memory, the system memory on the computer is shared among 12 CPU cores, and this become a bottleneck when there are more than 8 BWA threads running at the same time.

## Conclusions

Here we present BarraCUDA, a next generation sequencing alignment software to perform mapping of sequencing reads to reference genomes using NVIDIA graphics cards. Being based on BWA, BarraCUDA can perform gapped alignment with gap extensions and supports mappings for single- and paired-end reads with comparable alignment accuracies. BarraCUDA also generates alignments in the SAM format for compatibility with downstream data analysis applications.

Due to the limited amount of on-board memory and the tremendous number of threads to handle concurrently, a memory efficient DFS approach was used to perform inexact matches. Although DFS is not as time efficient as BFS utilized in BWA, BarraCUDA still offers a throughput of 6X the speed of a CPU core for gapped alignment and even faster when gap opening is disabled.

We also show here that multiple GPUs scales better than CPUs. A normal computer can easily take up 4 GPUs, meaning that using this test library as an example, a single-end alignment can be done in 5 min, which is twice the speed of a high-end 12-core workstation. Using 8X GPU, we can achieve an alignment speed 3X faster than a traditional computing node with 12 CPU cores, making GPU nodes a more favourable option, in terms of HPC environment, than using those with CPUs.

The software lays an important milestone in low-cost and energy efficient computing in bioinformatics using GPGPU. The software is still under active development and work is underway to further improve the program efficiency.

## Availability and requirements

Project Name: BarraCUDA

Project Home Page: http://seqbarracuda.sf.net

Operating System(s): Linux

Programming Language: C/C++, CUDA

Other Requirements: NVIDIA graphics cards with compute capability 1.3 or above, 768 MB VRAM, CUDA toolkit V4.0 or above

Licence: GNU GPL

Any Restrictions to use by non-academics: Nil

## Abbreviations

NGS: Next-generation sequencing; CUDA: Compute unified device architecture; SNP: Single-nucleotide polymorphism; BWT: Burrows-wheeler transform; DFS: Depth-first search; BFS: Breadth-first search; GPGPU: General purpose computing using graphics processing units; SM: Stream multi-processer; SIMT: Single-instruction multiple-thread; HPC: High-performance computing.

## Competing interests

The authors declare that they have no competing interests.

## Authors' contributions

BYHL and PK prepared the manuscript. BYHL, SL, PK and DL designed and implemented the software. MSC was involved in testing the software. GP provided the NVIDIA hardware for implementation. GP, GSHY and IM participated in initial feasibility analysis and were involved in the critical review of the manuscript. BYHL and GSHY supervised the overall progress of the project.

## Supplementary Material

Additional file 1 **Pseudo-code for BarraCUDA GPGPU alignment core and determining the memory workspace requirements for BWA's BFS and BarraCUDA's DFS alignment cores**. Click here for file
