# Supplementary material for: BarraCUDA - a fast short read sequence aligner using graphics processing units
Source: BMC Res Notes. 2012 Jan 13;5:27. doi: 10.1186/1756-0500-5-27 (PMC3278344; doi:10.1186/1756-0500-5-27)
Supplement: Additional file 1 — Pseudo-code for BarraCUDA GPGPU alignment core and determining the memory workspace requirements for BWA's BFS and BarraCUDA's DFS alignment cores. [file 1756-0500-5-27-S1.PDF]

Supplementary Material

Pseudo-code for BarraCUDA GPGPU alignment core

The following pseudo-code describes the new CUDA alignment core. BarraCUDA uses a data parallelism strategy and loads sequencing reads in batches onto the GPU to facilitate concurrent alignment computations. Each GPU thread contains a difference-bound depth-first search (DFS) agent that evaluates only one of the matching possibilities at a time on each node during search space traversal rather than storing all the possible matches in memory like BWA. For long sequencing reads, the program divides query into fragments of 32 bp and performs the alignment for each fragment in a consecutive manner.

Data parallelism

CUDA_alignment_core {

  Read *BWTs* from disk to GPU memory

  While there are unprocessed *sequencing reads* do {

    Read a batch of *n sequencing reads* (query) from disk to GPU memory

    Check if *sequencing read length* >= 38 bp (default threshold){
      //Divide query into fragments of 32bp and perform alignment with multiple GPU threads
      Launch split CUDA kernel with *n* threads {
       For each thread
          Calculate the *lower bound of the number of differences* for each base on the *read* up to 32bp //please refer to Li and Durbin 2009 for details
          Search_inexact_alignment for the first 32bp and **store** *partial alignments* in *GPU alignment store*
      }
    } **else** {
      //perform alignment with 1 GPU thread per read
      Launch normal CUDA kernel with *n* threads {
       For each thread
          Calculate the *lower bound of the number of differences* for each base on the *read*
          Search_inexact_alignment and **store** *alignments* in *GPU alignment store*
      }
    }

Data management for split CUDA kernel

```
Create partial alignment store
// a queue for storing partial alignments generated from the previous kernel

Do {
    Copy alignments from GPU alignment store to a temporary memory store on the
    host

    For each alignment in the temporary memory store do {
        Check if alignment is finished {
            Store alignment to the final alignment store)
        } else {
            //if it is a partial alignment
            Add partial alignment to partial alignment store
        }
    }
    Check if there still partial alignments in the partial alignment
    store {

        Select n x lowest-scored partial alignments from the store for the next
        kernel run

        Copy n x partial alignments to the GPU

        Launch CUDA kernel with n threads {
         For each thread
            Calculate the lower bound of the number of differences for the
            next 32 bp of the read
            Search_inexact_alignment for the next 32bp with data stored in
            the partial alignment store and store new alignments in GPU
            alignment store
    }

 } loop while there are still partial alignments in the partial alignment store

 For each of the reads from the final alignment store{
     Write alignments to the disk
 }

} //end of CUDA_alignment_core
```

Difference-bound depth-first search (DFS) agent for inexact alignments

Search_inexact_alignment {

Initializations
Check if it is a continuation of previous alignment, i.e. a *partial alignment* **exists**
{
Recall the *last aligned base position*
Recall *differences* and *suffix array boundaries*
} else{
//This is a new unaligned read
Set *last aligned base position* to 1
Set *suffix array* lower bound *k* to *0*
Set *suffix array* upper bound *l* to the size of *BWT* ,
}
Check if *base position* is within the *seed_length* → **set** *number of allowed differences* to the *number of allowed difference in the seed*

Check if *number of differences* > *number of allowed differences* → **exit**

Check if the DFS agent has **returned** to the *last aligned base position* → **exit**

Check if the DFS agent has reached the **last** *base* of the *query* {
Store *suffix array coordinates* and *differences* to the *GPU alignment store*
Mark as *finished*
}

Check if the DFS agent has reached the **end** of the *fragment* (32bp){
Store *suffix array coordinates* and *differences* to the *GPU alignment store*
Mark as *unfinished* and **store** the *last aligned base position*
}

For each *base* on the query, the agent **evaluates** only **one** of the following alignment possibilities:

Case 1. Exact match
Evaluate if there is an exact ***match** resulting in *k <= l* →
Search_inexact_alignment with next base and new *boundaries [k,l]*

Case 2. Mismatches
Substitute base with other bases and **evaluate if** there is a ***match** with *k <= l* → **record** differences and **Search_inexact_alignment** with next base and new *boundaries [k,l]*

Case 3. Indels
Shift base position (either by jumping to the next base or inserting {A,G, C,T}) and **evaluate if** there is ***match** with *k <= l* →**record** differences and **Search_inexact_alignment** with next base and new *boundaries [k,l]*

Evaluate if there is no more matches above for the *current* base -> **return** to **previous** *base position* and **evaluate** other possibilities
}

*** Please refer to Li and Durbin 2009 for details about calculating a match using FM-index**

Calculating the Memory Workspace Required in BWA's BFS and BarraCUDA's DFS alignment core

BWA's BFS alignment core

BWA BFS alignment core uses a heap-like memory structure to store all the partial alignment matches as it traverses the search space. The program allocates space for the memory store dynamically at run time and expands the allocation when required. The default ceiling is set at 2,000,000 partial hit entries as a compromise between speed and accuracy, and can be changed using the '-m' option. When aligning long sequence reads, it is not unusual that the default ceiling is reached from time to time.

Given that each of the partial match entries contains

5 x 4 bytes (32 bit) = 20 bytes of data

The maximum workspace required for BWA for each sequence read

= 2,000,000 * 20 bytes = 40 Mbytes

BarraCUDA's DFS alignment core

BarraCUDA only stores the data of partial matches (nodes) that are on the path of the search agent and the space requirement is much less than BWA at $O(n)$ where n is the length of the sequence read to be aligned. Assuming the sequence read is 100bp long, the memory requirement is therefore

= 100 * 20 bytes = 2 Kbytes

which is 20,000 fold less than BWA
